# Effect of Light-Dark Cycle Misalignment on the Hypothalamic-Pituitary-Gonadal Axis, Testicular Oxidative Stress, and Expression of Clock Genes in Adult Male Rats

**DOI:** 10.1155/2020/1426846

**Published:** 2020-11-06

**Authors:** Amira Moustafa

**Affiliations:** Department of Physiology, Faculty of Veterinary Medicine, Zagazig University, Zagazig 44519, Egypt

## Abstract

This study investigated the influence of circadian misalignment on the male reproductive system. Adult Sprague-Dawley male rats were exposed to prolonged light (20 h light : 4 h dark) or prolonged darkness (4 h light : 20 h dark) for 12 consecutive weeks. The somatic index of seminal vesicles and prostates increased due to prolonged light exposure. Sperm count and motility were enhanced solely by prolonged light exposure, whereas the percentage of sperm abnormalities was reduced by both prolonged light and darkness. The serum levels of reproductive hormones (follicle-stimulating hormone, luteinizing hormone, testosterone, and prolactin) were elevated, and the estradiol level was reduced by long-term light and dark exposure. Testicular total antioxidant capacity and antioxidant enzyme activities were improved, and lipid peroxidation was inhibited following chronic exposure to light or dark. Chronic light exposure increased, but chronic darkness decreased, testicular nitric oxide production. The mRNA expression of the hypothalamic and testicular clock genes including *PER1-2*, *CRY1-2*, *BMAL1*, *CLOCK*, and *Rev-Erbα* was altered by circadian disruption. Prolonged light exposure decreased the levels of thyroid hormones and suppressed the mRNA expression of adiponectin receptors 1 and 2. The immunohistochemical expression of proliferating cell nuclear antigen was decreased only by chronic darkness. The present study thus provides new insights into the physiological changes associated with long-term exposure to light or darkness, in which the expression levels of various clock gene mRNAs are modulated, reproductive hormones are increased, and the antioxidant enzyme system is ameliorated as mechanisms of adaptation to chronic circadian disruption.

## 1. Introduction

Reproductive health influences quality of life. The testis is a vital organ for hormone synthesis and metabolism. The hypothalamic-pituitary-testicular axis governed the process of spermatogenesis with the gonadotropin-releasing hormone (GnRH) as a crucial hypothalamic signal to the anterior pituitary. GnRH1 and its receptors are not only expressed in the brain but also in rodent testis, which control the production of high-quality sperm [1]. GnRH releases the luteinizing hormone (LH) and follicle-stimulating hormone (FSH) from the anterior pituitary, which control testicular functions and spermatogenesis [2, 3]. In mammals, testicular growth and regression are photoperiod dependent, meaning they are determined by the endogenous circadian secretion of melatonin [4].

Endogenous biological rhythms regulate multiple physiological and behavioral processes in mammals that are essential for proper reproduction; their misalignment provokes reproductive disorders. Endogenous circadian rhythms have been identified for melatonin, cortisol, thyroid-stimulating hormone (TSH), and to a lesser extent prolactin [5]. Clock genes within the suprachiasmatic nucleus (SCN) of the hypothalamus control such rhythms. Approximately 10 genes have been identified that are fundamental to cellular rhythmicity: *PER1*, *PER2*, *PER3*, *CLOCK*, *BMAL1*, *CRY1*, *CRY 2*, *DEC1*, *DEC2,* and *Rev-Erbα*, with *BMAL1* and *CLOCK* being the central genes [6]. In the main loop, the transcription factors *CLOCK* and *BMAL1* stimulate the expressions of *PER1–3* and *CRY1-2* [7], which in turn suppress *CLOCK/BMAL1* transcription and expression [8] and shut down their own transcription. *CLOCK/BMAL1* also controls the rhythmic expression of *Rev-Erbα*. *CLOCK* genes exist in central and peripheral reproductive tissues [9], and temporal coordination within and between tissues is vital for reproduction.

The ability of an organism to adapt to changes in its environment is an essential survival mechanism. Alteration of the circadian rhythm is common in modern society. Therefore, the present study investigated the effect of circadian misalignment due to prolonged light or prolonged dark exposure on male reproductive physiology by evaluating sperm concentration, motility, and abnormalities and possible underlying mechanisms of these through measurement of the levels of reproductive hormones, testicular antioxidant capacity, proliferating cell nuclear antigen (PCNA), and expression of clock genes.

## 2. Materials and Methods

### 2.1. Animal

Thirty mature male Sprague-Dawley rats were obtained from the laboratory animal unit at the Faculty of Veterinary Medicine, Zagazig University, and they were permitted to acclimate at an ambient temperature of 22°C and under a 12 h : 12 h light-dark cycle for 2 successive weeks. All experiments were approved by the institutional Animal Care and Use Committee of the Faculty of Veterinary Medicine, Zagazig University (Approval No. ZU-IACUC/2/F/139/2019).

### 2.2. Experimental Design

The rats were randomly assigned to three groups (10 rats per group) as indicated previously [10] with slight modifications: the control group (12 h : 12 h light-dark cycle), the prolonged light exposure group (20 h : 04 h light-dark cycle), and the prolonged dark exposure group (04 h : 20 h light-dark cycle). Rats were kept under different light-dark regimes for 12 consecutive weeks. At the end of the exposure period, rats were fasted overnight with free access to water, after which they were humanely euthanized through exsanguination. Blood samples were collected, and the sera were separated and stored at −20°C for subsequent measurements.

### 2.3. Weighing and Histological Analysis of Reproductive Organs

Different reproductive organs (namely, the testes, epididymides, seminal vesicles, and prostates) were dissected, freed of fat and connective tissues, and then weighed using an electronic scale. Somatic indices for reproductive organs were calculated using the following formula: somatic index = (weight of the tissue (g)/body weight of the animal (g)) × 100. One testis, epididymis, seminal vesicle, and prostate were taken from each rat, fixed in 10% buffered formalin, embedded in paraffin, and sectioned. Serial sections, each 5°*µ*m thick, were stained with hematoxylin and eosin and then examined and photographed with a digital camera attached to a photomicroscope (Olympus, Tokyo, Japan).

### 2.4. Semen Analysis

Immediately after the rats were killed, the cauda epididymis of all rats from all three groups were dissected and cut into small pieces in physiological saline at 37°C; the resultant suspension was used to evaluate the motility rate, sperm count, and percentage of sperm abnormalities. Sperm motility was assessed on prewarmed glass slides at 40 ×. Sperm count was analyzed as previously reported [11]. In brief, semen sample was diluted 5 times (v/v), and spermatozoa were counted using a hemocytometer chamber slide. The number of spermatozoa was calculated as follows: total number of sperms in 4 squares × 2500 × dilution factor. The percentage of sperm abnormalities was determined using eosin- and nigrosin-stained sections, with 100 spermatozoa examined through a 100 × immersion oil objective.

### 2.5. Hormone Measurement

Measurements of different hormones were taken in serum samples, using commercially available rat enzyme-linked immunosorbent assay (ELISA) kits, according to the manufacturer's protocol. Rat testosterone, estradiol (E2), prolactin, corticosterone, and TSH assay kits were obtained from Cusabio Biotech Company (Wuhan, China). Rat LH and FSH ELISA kits were purchased from Kamiya Biomedical Company (Seattle, USA). Rat triiodothyronine (T3) and thyroxine (T4) ELISA kits were obtained from MyBioSource (San Diego, CA, USA). The intraassay and interassay precisions for all utilized ELISA kits were <15%.

### 2.6. Measurement of Oxidative Stress

Testicular homogenates were used to evaluate antioxidant enzyme activities, nitric oxide (NO) concentrations, and lipid peroxidation levels (malondialdehyde; MDA activity). Superoxide dismutase (SOD) was measured using commercial ELISA kits (Cusabio Biotech Co., Ltd., Wuhan, China), according to the manufacturer's protocol. Catalase (CAT), glutathione peroxidase (GPx), and total NO levels were estimated using rat ELISA kits (MyBioSource, San Diego, CA, USA), according to the manufacturer's instructions. The levels of lipid peroxidation were determined using MDA assay kits (Elabscience Biotechnology Co., Ltd., Wuhan, China). Total antioxidant capacity (TAC) in semen and testicular homogenate was estimated using Cell Biolabs OxiSelect assay kits (San Diego, Inc., CA, USA), according to the manufacturer's instructions.

### 2.7. Quantitative Real-Time Reverse Transcription Polymerase Chain Reaction

TRIzol (Invitrogen; Thermo Fisher Scientific, Inc.) was used to extract total RNA, according to the manufacturer's instructions. The quality of the extracted RNA was assessed using the NanoDrop ND-1000 spectrophotometer (NanoDrop Technologies, Wilmington, Delaware, USA). The total RNA was reverse transcribed using a cDNA synthesis kit (iNtRON Biotechnology Co., South Korea). Reaction mixtures were incubated in a Veriti 96-well thermal cycler (Applied Biosystems, Foster City, CA) for 60 min at 45°C, followed by 10 min at 85°C. The real-time reverse transcription polymerase chain reaction was conducted in an Mx3005P Real-Time PCR System (Agilent Stratagene, USA) using the SYBR Green PCR Master Mix (Enzynomics, Daejeon, Korea), according to the manufacturer's instructions. The PCR conditions were as follows: an initial denaturation at 95°C for 12 min, 40 cycles of denaturation at 95°C for 15 s, annealing at 60°C for 30 s, and extension at 72°C for 30 s. Quantitative PCR data were normalized using GAPDH mRNA levels as the internal standard. The relative changes in gene expression between different groups were calculated using the 2^ΔΔC*t*^ method [12], and data were expressed as a percentage relative to the GAPDH mRNA levels. Oligonucleotide primers (Table 1) were synthesized by Eurofins Genomics (Ebersberg, Germany).

### 2.8. PCNA Immunohistochemical Determination

Assessment of the immunohistochemical distribution of PCNA in rat testes was performed using an avidin-biotin-peroxidase method (Elite-ABC, Vector Laboratories, California). Briefly, 5 *µ*m thick paraffin sections were mounted on poly-L-lysine-coated slides and then deparaffinized, rehydrated, and washed; then, peroxidase activity was inhibited using 0.3% H_2_O_2_. Subsequently, slides were incubated with a blocking solution (1% bovine serum albumin in phosphate buffered saline (PBS)) at room temperature for 10 min. After washing with PBS, sections were incubated for 1 h with biotinylated mouse PCNA primary antibody (1 : 100, DAKO Japan Co, Ltd, Tokyo, Japan), and slides were incubated with streptavidin-peroxidase at room temperature for 10 min. Diaminobenzidine (DAKO, USA) was used as a chromogen. Finally, the sections were washed and counterstained with hematoxylin. The primary PCNA antibody was omitted in the negative control slides. All slides were viewed and photographed with a digital camera attached to a photomicroscope (Olympus, Tokyo, Japan). The stained areas were analyzed with the ImageJ software package, and data were expressed as a percentage of the area fraction as indicated previously [13].

### 2.9. Statistical Analysis

Data are presented as mean values ± standard error of the mean. Whether there were significant differences between groups was investigated using one-way analysis of variance, and Student's *t*-test for independent groups was utilized to estimate the differences between two groups. *P* < 0.05 was considered statistically significant.

## 3. Results

### 3.1. Effect of Changing the Light-Dark Cycle Regime on Sperm Characteristics

Sperm cell concentration, sperm motility, and sperm morphology were assessed after 12 weeks of exposure to different light-dark cycle regimes. Long-term exposure to light significantly increased sperm count in male rats, whereas dark exposure exhibited no significant effect (Table 2). Moreover, sperm motility was significantly improved only by prolonged light exposure. However, the percentage of morphologically abnormal sperm was significantly reduced by both prolonged light and prolonged dark exposure when compared with the control group (Table 2).

### 3.2. Effect of Changing the Light-Dark Cycle Regime on Reproductive Organs Somatic Indices and Corticosterone Concentrations

**Figure 1:**
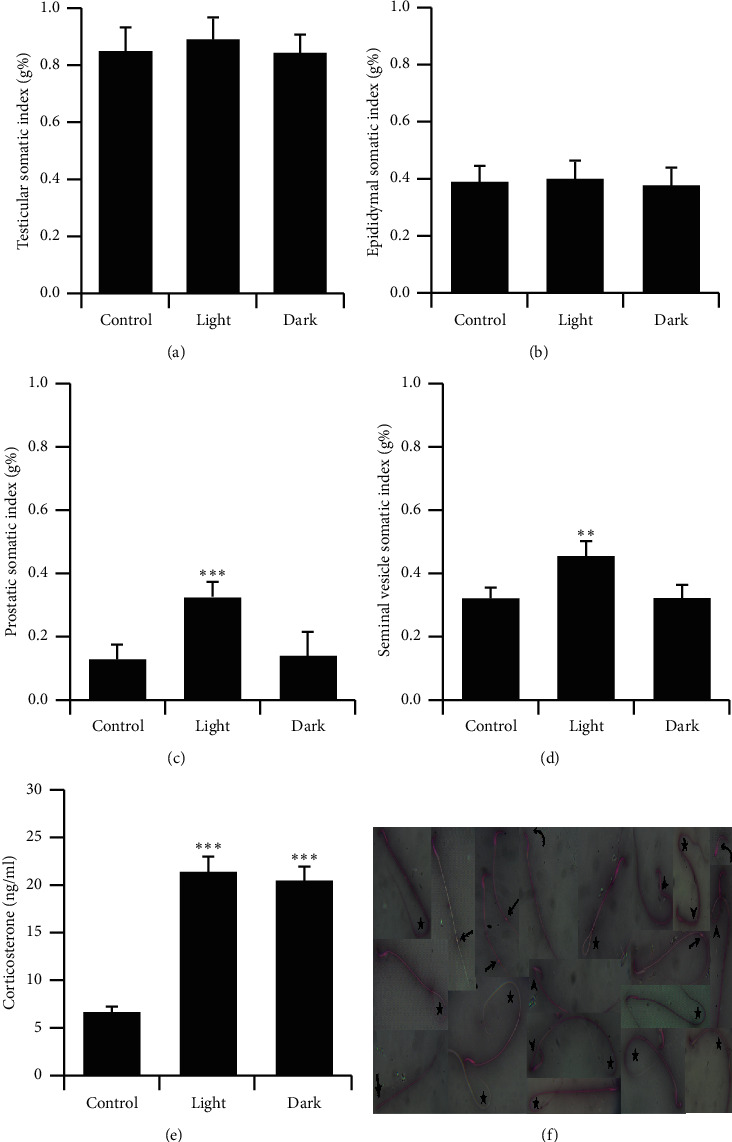
Relative changes in the reproductive organs weight and corticosterone levels. Somatic index of (a) testes, (b) epididymides, (c) prostates, and (d) seminal vesicles of male rats exposed to prolonged light or prolonged darkness (*n* = 8–10 rats). (e) Serum corticosterone concentrations (*n* = 8–10 rats per group) (ng/ml). ^*∗∗*^*P* ≤ 0.01 and ^*∗∗∗*^*P* ≤ 0.001*vs* the control group by Student's *t*-test. (f) Photomicrographs illustrating various sperm abnormalities. Asterisks, tail defects; arrows, protoplasmic droplets; arrow heads, midpiece defects; curved arrows, head defects; thick arrow, detached tail.

Prolonged light or dark exposure was not associated with any significant difference in the testicular and epididymal somatic indices (Figures 1(a) and 1(b)). However, the somatic indices of the seminal vesicles and prostates were significantly elevated following prolonged light exposure (Figures 1(c) and 1(d), *P* < 0.001and *P* < 0.01 by Student's *t*-test, respectively). The circulating levels of corticosterone were significantly elevated in rats exposed to prolonged light and prolonged dark (Figure 1(e), *P* < 0.001 by Student's *t*-test). Representative photomicrographs of sperm morphology abnormalities are shown in Figure 1(f).

### 3.3. Effect of Changing the Light-Dark Cycle Regime on the Level of Reproductive Hormones

**Figure 2:**
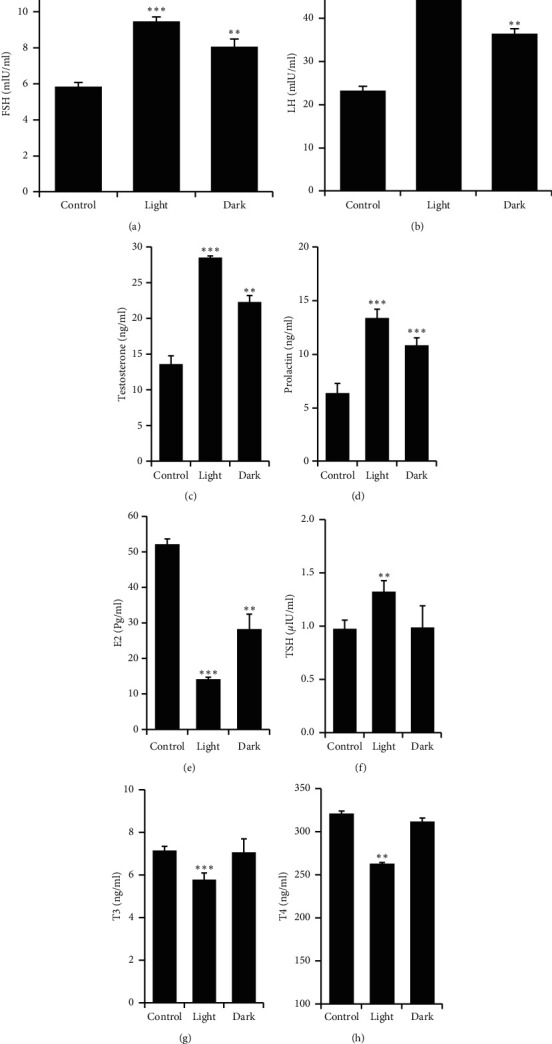
Changes in the levels of reproductive and thyroid hormones. Serum levels of (a) FSH (mIU/ml), (b) LH (mIU/ml), (c) testosterone (ng/ml), (d) prolactin (ng/ml), (e) E2 (pg/ml), (f) TSH (*µ*IU/ml), (g) T3 (ng/ml), and (h) T4 (ng/ml) in male rats after prolonged light or prolonged dark exposure for 12 consecutive weeks. Values are expressed as means ± SEM (*n* = 8–10 rats). ^*∗∗*^*P* ≤ 0.01 and ^*∗∗∗*^*P* ≤ 0.001*vs* the control group by Student's *t*-test.

The hypothalamic-pituitary-gonadal (HPG) axis controls the physiology of reproduction in mammals. Therefore, serum levels of FSH, LH, testosterone, prolactin, and E2 were estimated. Changing the light-dark cycle regime either through prolonged light or prolonged dark exposure significantly increased the levels of FSH, LH, testosterone, and prolactin (Figures 2(a)–2(d)). However, the levels of E2 were markedly decreased following long-term exposure to light or dark (Figure 2(e), *P* < 0.001 and *P* < 0.01 by Student's *t*-test, respectively).

### 3.4. Effect of Changing the Light-Dark Cycle Regime on Thyroid Hormones

Circulating levels of TSH were significantly increased following prolonged light exposure (Figure 2(f), *P* < 0.01 by Student's *t*-test), whereas serum levels of T3 and T4 significantly decreased (Figures 2(g) and 2(h), *P* < 0.01 and *P* < 0.001 by Student's *t*-test, respectively). Prolonged dark exposure had no significant effects on thyroid hormones.

### 3.5. Effect of Changing the Light-Dark Cycle Regime on Antioxidant Enzyme Activities, Lipid Peroxidation, NO Production, and TAC

**Figure 3:**
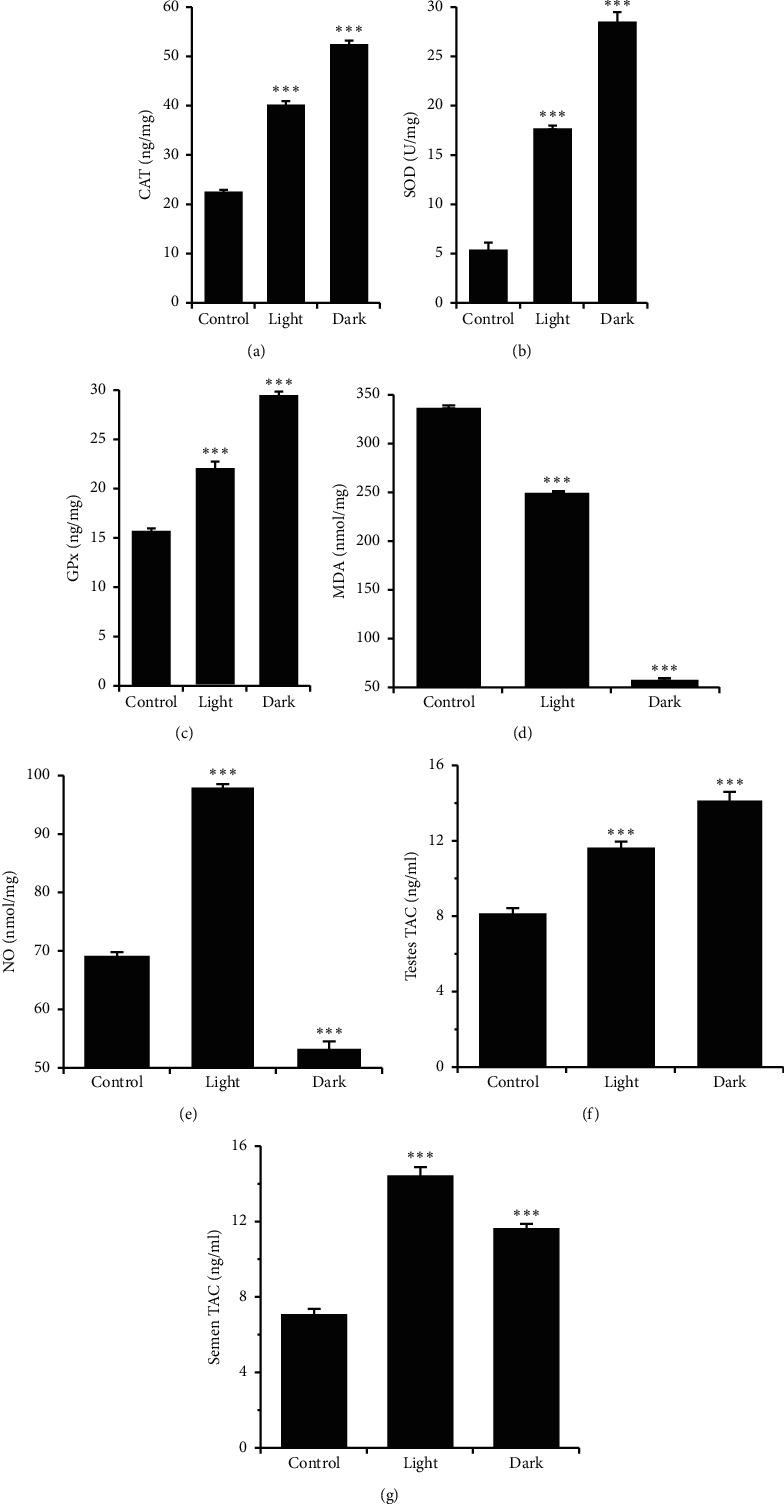
Changes in the levels of antioxidant enzymes, MDA, NO, and TAC. Testicular levels of (a) CAT (ng/mg), (b) SOD (U/mg), (c) GPx (ng/mg), (d) MDA (nmol/mg), (e) NO (nmol/mg), (f) TAC, and (g) semen TAC levels (ng/ml) in male rats following exposure to prolonged light or prolonged darkness for 12 consecutive weeks. Data are presented as means ± SEM (*n* = 8–10 rats). ^*∗∗∗*^*P* ≤ 0.001*vs* the control group by Student's *t*-test.

The activities of the endogenous enzymatic antioxidants, namely, SOD, CAT, and GPx, were investigated. Long-term exposure to light or dark significantly increased the testicular levels of CAT, SOD, and GPx (Figures 3(a)–3(c), *P* < 0.001 by Student's *t*-test). However, testicular levels of MDA, the lipid peroxidation marker, were significantly reduced by prolonged light exposure and substantially inhibited by prolonged dark exposure (Figure 3(d); *P* < 0.001 by Student's *t*-test).

The levels of NO in the testicular tissues were significantly elevated by prolonged light exposure and markedly reduced by prolonged dark exposure compared with the control group (Figure 3(e); *P* < 0.001 by Student's *t*-test).

Testicular and seminal TAC were significantly increased following prolonged light and prolonged dark exposure compared with the control group (Figures 3(f) and 3(g), *P* < 0.001 by Student's *t*-test).

### 3.6. Effect of Changing the Light-Dark Cycle Regime on Gene Expression

**Figure 4:**
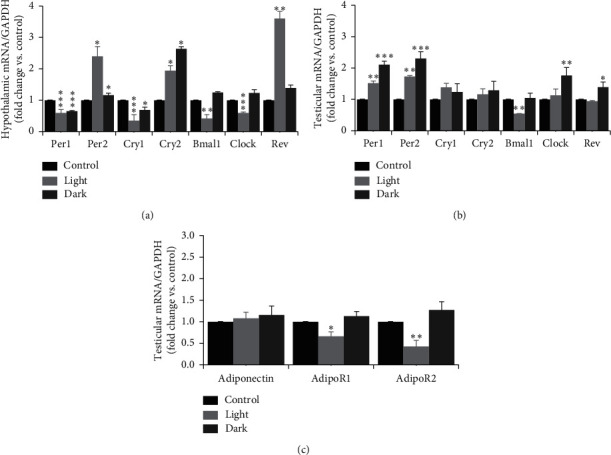
Q-RT-PCR analysis of hypothalamic and testicular genes expression in male rats. Clock genes mRNA expression in (a) the hypothalamus and (b) testes of male rats following exposure to prolonged light (bright gray) or prolonged dark (dark gray) for 12 consecutive weeks. (c) Testicular mRNA expression of adiponectin and adiponectin R1 and R2. Data are expressed as means ± SEM (*n* = 8–10 rats). ^*∗*^*P* ≤ 0.05, ^*∗∗*^*P* ≤ 0.01, and ^*∗∗∗*^*P* ≤ 0.001.

All clock genes examined in the present study (*PER1*, *PER2*, *CRY1*, *CRY2*, *BMAL1*, *CLOCK*, and *Rev-Erbα*) were expressed in the hypothalamus and testes of male rats (Figures 4(a) and 4(b)). In the hypothalamus, prolonged light exposure upregulated the relative mRNA expression of *PER2*, *CRY2*, and *Rev-Erbα* (Figures 4(a), *P* < 0.05 and *P* < 0.01 by Student's *t*-test, respectively) and downregulated the mRNA expression of *PER1*, *CRY1*, *BMAL1*, and *CLOCK* (Figure 4(a), *P* < 0.001 and *P* < 0.01 by Student's *t*-test). However, prolonged dark exposure increased the mRNA expression levels of *PER2* and *CRY2* (Figure 4(a), *P* < 0.05 by Student's *t*-test) and decreased the expression levels of *PER1* and *CRY1* (Figure 4(a), *P* < 0.001 and *P* < 0.05 by Student's *t*-test, respectively).

In the testicular tissues, the relative mRNA expression of *PER1* and *PER2* genes increased, whereas *BMAL1* mRNA expression decreased following prolonged light exposure (Figure 4(b), *P* < 0.01 by Student's *t*-test). The mRNA expression levels of *PER1*, *PER2*, *CLOCK*, and *Rev-Erbα* were upregulated by prolonged dark exposure (Figure 4(b), *P* < 0.001, *P* < 0.01, and *P* < 0.05 by Student's *t*-test, respectively). Testicular adiponectin mRNA expression levels exhibited no changes following different light-dark cycle patterns. However, the mRNA expression levels of adiponectin receptors 1 and 2 were significantly inhibited by prolonged light exposure (Figure 4(c), *P* < 0.05 and *P* < 0.01 by Student's *t*-test, respectively). Prolonged dark exposure had no significant effects on adiponectin and its receptors.

### 3.7. Effect of Changing the Light-Dark Cycle Regime on PCNA Immunohistochemistry

**Figure 5:**
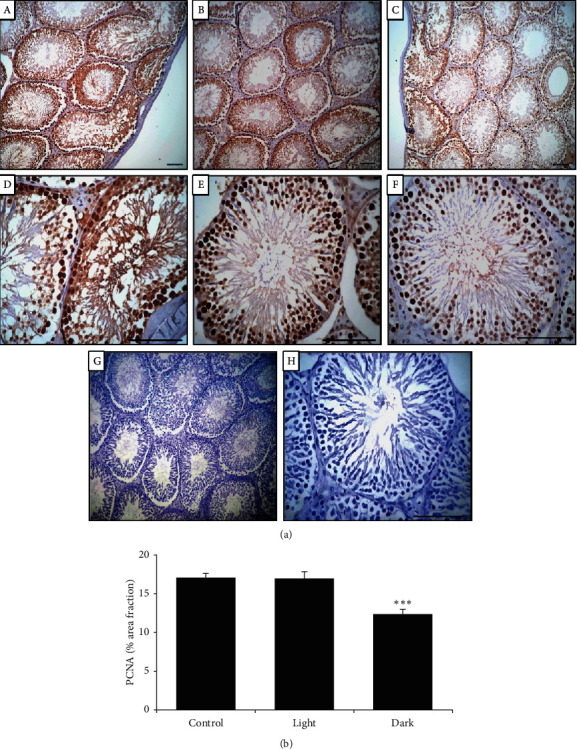
Immunohistochemistry for PCNA localization in rat testes. (a) PCNA immunohistochemistry in testes of (A, D) control, (B, E) prolonged light, (C, F) prolonged dark-exposed groups, and (G, H) negative control. Scale bar = 250 *µ*m. (b) Percentage of area fraction for PCNA-positive cells. ^*∗∗∗*^*P* ≤ 0.001*vs* the control group by Student's *t*-test.

For rats exposed to normal light-dark cycle patterns or long-term light, substantial PCNA immunoreactive positive cells were present in the seminiferous tubules, and reactions were prominent in the spermatogonia, primary spermatocytes, secondary spermatocytes, and spermatids (Figures 5(a) and 5(b)). However, the testes of rats exposed to prolonged darkness demonstrated weak PCNA immunostaining compared with the controls, and expression was absent in the spermatids (Figure 5(a)). Moreover, the percentage of PCNA-positive germinal cells had significantly declined (Figure 5(b)).

### 3.8. Effect of Changing the Light-Dark Cycle Regime on Morphology of Reproductive Organs

**Figure 6:**
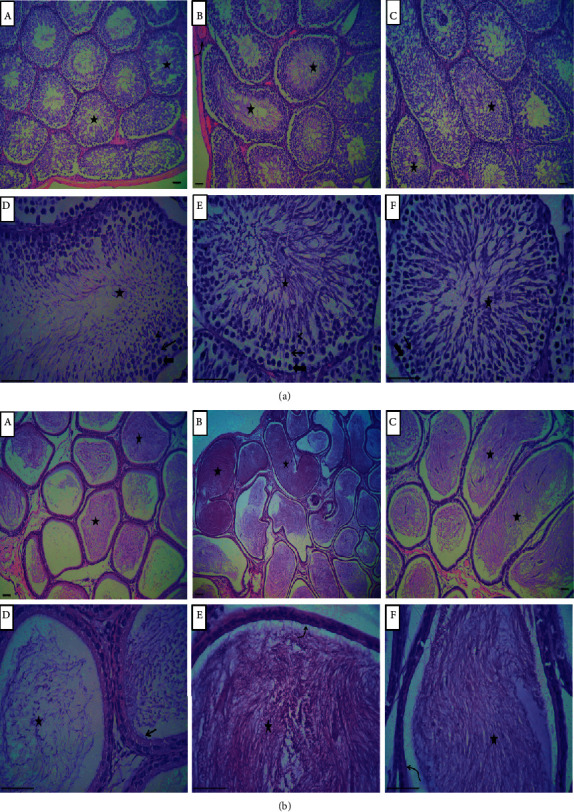
Histological changes in the testes and epididymides. (a) Rat testes. (A, D) Testes of the control group showing normal histomorphology of spermatogonia (thick arrow), spermatocytes (arrow), spermatid (arrow head), and mature sperms within lumen of seminiferous tubules (asterisks). (B, E) Testes of rats after prolonged light exposure illustrating normal spermatogonia (thick arrow), hyperactive spermatogenesis (arrow), and spermiogenesis (arrow head) with large number of sperms within lumina (asterisks) and normal testicular blood vessels (curved arrow). (C, F) Testes of rats after prolonged dark exposure showing normal spermatogonia (thick arrow), spermatocytes (arrow), and numerous numbers of mature sperms within most seminiferous tubules lumina (asterisks). (b) Rat epididymis. (A, D) Photomicrograph of epididymis of the control group showing stored sperms within tubular lumina (asterisks) and pseudostratified columnar epithelium with stereocilia lining epithelium (arrow). (B, E) Epididymis of the prolonged light-exposed group revealing clumps of spermatozoa within epididymal lumina (asterisks) with pseudostratified columnar epithelium lining tubules (curved arrow). (C, F) Photomicrograph of epididymal tubules in the prolonged dark-exposed group showing low columnar to cuboidal lining epithelium (curved arrow) and numerous numbers of spermatozoa within epididymal lumina (asterisks). Scale bar = 250 *µ*m.

The histological structure of the testes of the control group exhibited normal histomorphological structures of spermatogonia, with preserved spermatogenesis and the lumen of the seminiferous tubules filled with mature sperm. Following prolonged light exposure, testes exhibited hyperactive spermatogenesis and spermiogenesis. The seminiferous tubules were characterized by the presence of a large number of sperm within the lumina. The testes of rats exposed to prolonged dark exhibited normal germinal epithelial lining the seminiferous tubules, including spermatogonia, spermatocytes, spermatids, spermatozoa, and Sertoli cells. Spermatogenesis processes were active as indicated by high numbers of sperm within the lumina of most seminiferous tubules (Figure 6(a)).

The epididymides of rats in the control group had normal stored mature sperm or spermatids within the epididymal tubules, but the epididymides following prolonged light exposure exhibited a compact organization of convoluted tubules and clumps of spermatozoa within the epididymal lumina. In rats exposed to prolonged dark, the epididymal tubules exhibited low columnar- to cuboidal-lined epitheliums with stereocilia and numerous spermatozoa within the epididymal lumina (Figure 6(b)).

**Figure 7:**
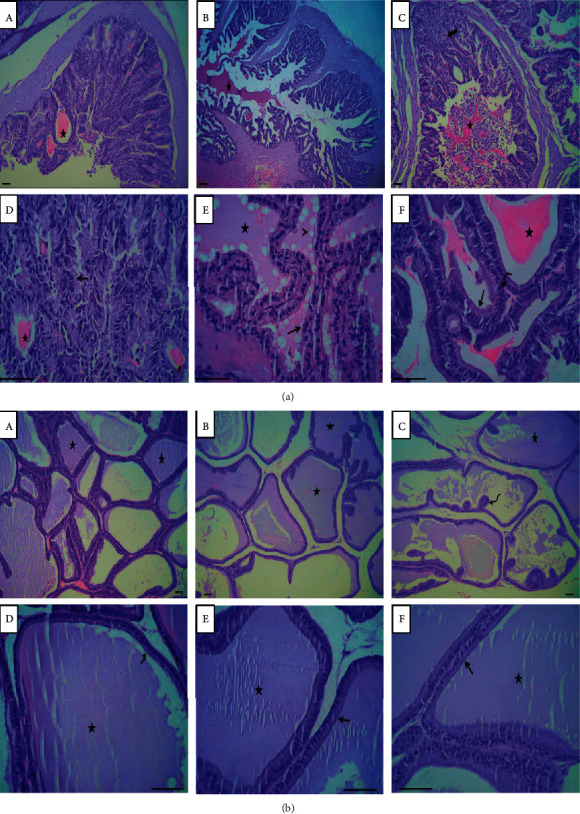
Histological changes in the seminal vesicles and prostates. (a) Rat seminal vesicles. (A, D) Photomicrograph of seminal vesicle of control rats showing columnar or pseudostratified columnar epithelial lining glands (arrow) and acidophilic secretory products within the glands lumina (asterisks). (B, E) Photomicrograph of seminal vesicle following prolonged light exposure illustrating pseudostratified columnar epithelial lining mucosa (arrow) and eosinophilic secretion (asterisks) with its lipoidal contents (arrow head) within glandular lumen. (C, F) Photomicrograph of seminal vesicle after prolonged dark exposure showing mild hyperplastic epithelial lining the glands (thick arrow) with columnar cells (arrow) and basal cells (curved arrow) beside accumulation of large amount of secretory materials within the glandular lumina (asterisks). (b) Rat prostates. (A, D) Photomicrograph of prostate of control rat showing cuboidal or columnar epithelial lining gland (arrow) and acidophilic secretion within the glandular lumina (asterisks). (B, E) Photomicrograph of rat prostate following prolonged light exposure showing eosinophilic inclusions in the glandular lumina (asterisks) with cuboidal or low columnar cells epithelial lining the gland (arrow). (C, F) Photomicrograph of rat prostate after prolonged dark exposure showing papillary projections of the mucosa into the lumen of the gland (curved arrow), simple columnar epithelial lining the secretory alveoli (arrow), and eosinophilic secretions within the lumina (asterisks). Scale bar = 250 *µ*m.

The seminal vesicles of the control group revealed branched, anastomosing folds of the mucosa, with a columnar or pseudostratified columnar epithelial lining of the glands. Acidophilic secretory products of the columnar cell were seen within the lumen of the seminal vesicles (Figure 7(a), A, D). Seminal vesicles of the group exposed to prolonged light exhibited complex papillary folds, and the lumens of the seminal vesicles were filled with their acidophilic secretion, with clear lipid droplets (Figure 7(a), B, E). Following prolonged dark exposure, the seminal vesicles exhibited convoluted folds in the mucosal lining of the glands. Mild hyperplasia was evident in some areas of the glandular tissue, with accumulation of secretory materials within the lumina (Figure 7(a), C, F).

The prostates of the rats exposed to the normal light-dark cycle had normal histomorphological structures, represented by a cuboidal or columnar epithelial lining of the gland and acidophilic secretions within the glandular lumina. However, the prostates of rats exposed to prolonged light had an irregular acinar shape, with villous projections into the lumen and the presence of eosinophilic inclusions within the acini lumina. The prostates of the prolonged dark-exposed rats included irregularly shaped secretory alveoli due to the papillary projections of the mucosa into the lumen of the gland, with eosinophilic secretions within their lumina. The prostates were lined by a simple columnar epithelium that changed to a transitional epithelium near the opening of the ducts into the urethra (Figure 7(b)).

## 4. Discussion

The circadian system permits biological processes to predict and acclimate to the 24 h light-dark cycle, which guarantees optimal physiological functioning. Therefore, circadian disruption may participate in the onset and development of disease. Understanding the effects and mechanisms through which the circadian system regulates the male reproductive system may help to improve the reproductive health of organisms.

Long photoperiods stimulate and short photoperiods inhibit reproductive development and gonadal functions in rats [14]. Moreover, a long photoperiod increases [15] and a short photoperiod decreases testicular weight and serum testosterone and hypophyseal LH levels [16, 17]. The hormones of the HPG axis enhance and support male sexual development and functions, and the homeostatic growth of adult male accessory glands is under the control of androgen and its receptors. Although the serum levels of FSH, LH, and testosterone were elevated by long-term exposure to light or dark in the present study (Figures 2(a)–2(c)), prolonged exposure to light solely increased the somatic indices of the prostate and seminal vesicle (Figures 1(c) and 1(d)). Increased weights of seminal vesicles and prostates may be due to increased fluid content that was confirmed through histological examination of both accessory sex organs (Figures 7(a) and 7(b)). Although the exact reason for this was unclear, light exposure along with testosterone may have a permissive effect that enhances the weight of the accessory sex organs by increasing the number, sensitivity, or both of testosterone receptors. However, such an assumption needs further investigation. An inhibition of spermatogenesis was associated with constant dark exposure in rats [18]. Furthermore, daily exposure to 16 h of darkness for 8 weeks was reported to induce testes regression and decrease serum testosterone concentrations in mice [19]. Because testosterone is a crucial factor in the process of spermatogenesis, the elevated concentrations of testosterone noted in the present study (Figure 2(c)) may explain the augmentation observed in the seminal parameters of sperm count, motility, and morphology (Table 2). The enhanced sperm count was further confirmed through histological examination of cauda epididymis (Figure 6(b)). Rats exposed to a short photoperiod showed significant reductions in reproductive organ mass and sperm viability, count, and motility [20], whereas wild rabbits demonstrated ameliorated male reproductive characteristics [21].

Testosterone has been shown to stimulate prolactin release in male rats [22]. Moreover, an increase in plasma testosterone levels has been reported to proceed through a rising level of prolactin [23], whereas prolactin increases testosterone synthesis through upregulation of LH receptors on Leydig cells [24]. Prolactin reaches its highest levels around the light-dark transition period [25]. In the present study, long-term exposure to light or dark raised the serum levels of prolactin (Figure 2(d)), which may help to retain the competency of Leydig cells [26].

Circulating androgens are the primary source of estrogens. The immature germ cells, spermatozoa, epididymal ducts, Leydig cells, and Sertoli cells are the main sites for E2 synthesis [27, 28]. E2 has both positive and negative impacts on testicular cells [29]. In the testes, E2 has been reported to control various features of spermatogenesis, such as proliferation, differentiation, survival, and apoptosis of germ cells [30]. Moreover, E2 influences sperm concentration, motility, and morphology [31] and is essential for the production of high-quality mature spermatozoa in human and animal models [32, 33]. In the current study, despite the elevated testosterone concentrations, the circulating levels of E2 were abolished by both prolonged light and prolonged dark exposure (Figure 2(e)), indicating that circadian misalignment may interrupt the conversion of testosterone to E2. It has been reported that excess E2 has inhibitory effects on FSH and LH and decreases serum testosterone concentrations [34]. Therefore, the increased circulating levels of FSH, LH, and testosterone reported herein may be attributed to circadian disruption-induced inhibition of E2 synthesis.

Oxidative stress has destructive effects on cellular biomolecules, thereby hindering their normal functions. Antioxidant enzymes such as SOD, CAT, and GPx are the primary bodily defense against reactive oxygen species (ROS). Oxidative stress has been shown to increase abnormal sperm, reduce sperm count, and induce fragmentation of sperm DNA, resulting in infertility. Moreover, lipid peroxidation-induced ROS is a key factor in testicular dysfunction [35]. Therefore, the existence of antioxidant compounds in the seminal plasma determines the health and fertility of the sperm. Twelve weeks of exposure to either light or dark significantly elevated testicular and seminal TAC. In addition, the testicular concentrations of CAT, SOD, and GPx were increased, and the levels of the lipid peroxidation indicator, MDA, decreased under prolonged light and prolonged dark exposure (Figure 3). SOD gene expression has been shown to oscillate with daily rhythmicity in the rat lung, intestine, and cerebellum [36]. Moreover, in the rat cerebral cortex, SOD activity was reported to peak in the dark phase, simultaneous with MDA [37]. However, GPx activity peaked in the opposite phase as lipid peroxidation [37]. CAT activity in the liver and kidneys of nocturnal mice was shown to peak in the middle of the dark phase [38], but its peak was at the beginning of the light phase in plasma samples of diurnal humans [39]. Continuous light has been demonstrated to abolish the SOD and CAT circadian rhythms [40]. The relatively high activities of the antioxidant enzymes seen in the current study might be a predictive homeostasis mechanism to maintain an ideal cellular redox state for physiological functions.

NO, which is synthesized by nitric oxide synthase (NOS), is an essential element of the biological clock and circadian rhythm mechanisms [41]. It participates in the adaptation to environmental lighting conditions [42]. NO is vital for the reproductive organs in physiological and pathophysiological circumstances [43]. The present study investigated the influence of circadian disruption on testicular NO production and found that NO was markedly increased following prolonged light exposure and significantly inhibited by prolonged dark exposure (Figure 3(e)). NOS activity has been shown to peak in the brain regions during the behaviorally active circadian phase and is implicated in sleep regulation [44]. However, melatonin has been shown to regulate NOS activity, with a suppressive effect on the expression of NOS reported in rodents [45]. Plasma melatonin is associated with a circadian rhythm with high levels at night [46], and thus, it is conceivable that the low concentrations of NO observed in the present study following prolonged dark exposure may be attributed to dark-induced melatonin production. The time course changes in NOS activity do not necessarily reflect the changes of NO concentrations because NO may be carried and stored by proteins, and thus, NO may be increased during the rest period.

Seasonal rhythms in male reproductive functions have been reported, and clock genes control such rhythmicity [47]. Therefore, understanding the impact of circadian misalignment on hypothalamic and expression of testicular clock genes is crucial. Environmental light is the main stimulus of synchronization of the central clock in the SCN [48]. The data from the present study demonstrate increases in the hypothalamic mRNA levels of *PER2*, *CRY2*, and *Rev-Erbα* and decreases in the expression levels of *PER1*, *CRY1*, *BMAL1*, and *CLOCK* genes by prolonged light exposure. However, hypothalamic *PER2* and *CRY2* mRNA levels were upregulated, and the *PER1* and *CRY1* mRNA levels downregulated by prolonged dark exposure (Figure 4(a)). In the SCN of rats, *PER1*, *CRY1*, and *BMAL1* mRNA oscillated to a greater extent under short photoperiods [49]. Light at night triggers the expression of *PER1* and *PER2* but not of the other clock genes [50]. In nocturnal rodents' SCN, light at the end of the dark phase increases mRNA transcription of *PER1*, whereas light at the beginning of the dark phase delays the offset of *PER2* and thus delays the clock [51]. The photoperiod does not affect *BMAL1* or *CLOCK* mRNA in the SCN [52]. However, the fluctuation in *BMAL1* mRNA expression timing might be linked to the change in *PER2* production that was reported to be modified by the photoperiod [53]. In contrast to the hypothalamus, the testes do not display the apparent rhythmicity of clock gene expression [54]. The expression of PER1 and PER2 proteins in mouse tissue exhibited no change across the 24 h period. Furthermore, *BMAL1*, which controls the rhythmic expression of *PER1*, showed a nonrhythmic pattern in the testis [54]. In addition, the clock genes *PER2* and *CRY1* expressed nonrhythmic patterns in developing sperm and Leydig cells [55]. In the present study, testicular *PER1* and *PER2* mRNA expression levels were elevated following prolonged light and prolonged dark exposure, mRNA levels of *CLOCK* and *Rev-Erbα* genes were decreased by prolonged darkness, and *BMAL1* expression was only decreased by prolonged light exposure (Figure 4(b)). In Syrian hamsters, exposure to continuous darkness was shown to induce gonadal regression and changes in testicular clock gene expression patterns. Under such circumstances, the expression of the *PER1* transcripts became arrhythmic, whereas the expression of *BMAL1* remained entirely rhythmic [56]. The overall changes in clock genes expression found in the current study may reveal adaptation to the chronic alterations in the external environment.

Adipokines act on hypothalamic GnRH-expressing neurons and modulate GnRH-stimulated synthesis of LH, which is the principle regulator of testosterone synthesis [57]. Adiponectin is well known to be produced primarily from the adipose tissue, but it has also been shown to be synthesized from other tissues, such as the brain, pituitary, and testes [58, 59]. The target tissues for adiponectin express the adiponectin receptors AdipoR1 and AdipoR2 that bind with different affinities to adiponectin [60]. AdipoR1 was shown to be expressed in the epithelium of the seminiferous tubules, where it is involved in the regulation of spermatogenesis, whereas AdipoR2 was present on the surface of Leydig cells and is vital for testosterone synthesis [59]. Both types of adiponectin receptor are also located in the spermatozoa [59]. Because adiponectin has a fundamental role in the male HPG axis and regulation of steroidogenesis, the effects of circadian disruption on testicular adiponectin, *AdipoR1*, and *AdipoR2* mRNA expressions were examined. Long-term exposure to light significantly downregulated *AdipoR1* and *AdipoR2* mRNA expression levels (Figure 4(c)). In the testes, LH is the principle regulator of the adiponectin gene expression, and a high plasma concentration of LH increases the expression of adiponectin in adult rats [59]. Because testosterone production depends on LH activity, a positive correlation between adiponectin levels and testosterone synthesis was demonstrated [61]. Another factor that modifies testicular expression of the adiponectin gene is the thyroid hormone, whereas thyroxine upregulates adiponectin mRNA levels in the testis [59]. Moreover, the expression level of the adiponectin receptors gene was shown to correlate positively with thyroid hormone concentrations [62]. In the current study, prolonged light exposure decreased the concentrations of T3 and T4 (Figures 2(g) and 2(h)), whereas prolonged exposure to darkness had no effect on the circulating levels of thyroid hormones. This may explain, in part, the decreased mRNA expression levels of both *AdipoR1* and *AdipoR2* following prolonged light exposure but not prolonged darkness.

Circadian regulation of cell division genes has been reported in mice [63]. The expression of cell cycle-related genes is largely affected by clock mutation, with the DNA repair process reported to be under circadian control [64]. PCNA, as a cell cycle regulatory protein marker, is engaged in DNA replication, nucleic acid metabolism, and RNA transcription. PCNA is also involved in the proliferation and differentiation of spermatogonia in the testis [65], and smooth spermatogenesis is associated with positive expression of PCNA. Therefore, fluctuations in PCNA expression are used as a marker to evaluate spermatogenic cell proliferation. To date, few reports have evaluated the influence of changing the light-dark cycle on the expression of PCNA. The present study found positive PCNA expression throughout the testicular tissue, although immunohistochemical expression declined following prolonged dark exposure (Figure 5). Melatonin, the darkness hormone, has been reported to be an antiproliferative agent that decreases PCNA expression in a hormone-dependent manner in vivo and in vitro in mice prostate tumors [66] and in female rat ovarian cells [67]. Accordingly, darkness-induced melatonin production may have contributed to the observed decrease in the expression of PCNA.

## 5. Conclusion

**Figure 8:**
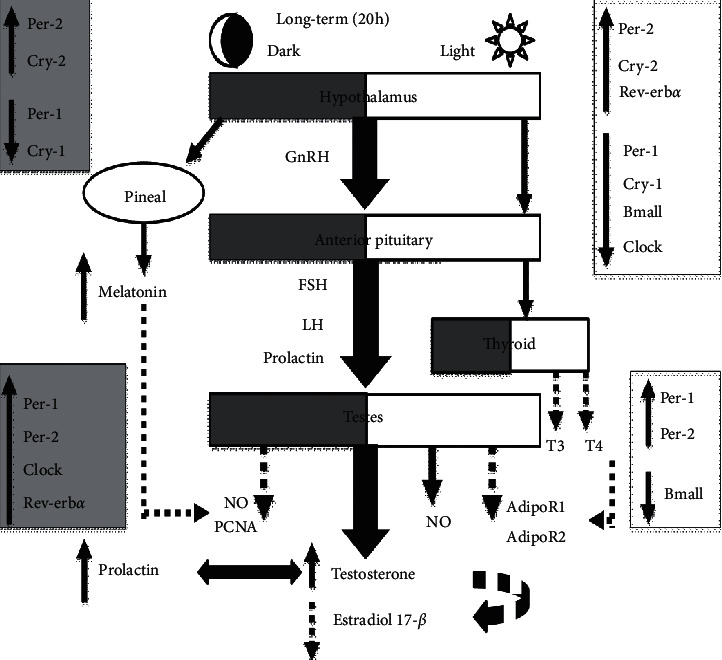
Schematic diagram illustrating circadian disruption-induced hormonal and genes expression changes in male rats. Solid lines indicate stimulation, while dashed lines indicate inhibition.

The present study indicates for the first time that increased production of reproductive hormones and amelioration of the antioxidant enzyme system may be adaptive responses to long-term exposure to light or darkness; their effects may be attributed to how they alter the expression of hypothalamic and testicular clock genes (Figure 8).

## Figures and Tables

**Table 1:** List of primer sequences used in PCR.

Gene	Forward primer (5ˊ–3ˊ)	Reverse primer (5ˊ–3ˊ)	Accession number	Product size (pb)
PER1	AGCAGAGGGCGGGTCCAGTT	TTATTGGCCAGGGCGAGCGG	NM_001034125.1	133
PER2	CGCACACGCAACGGGGAGTA	AACGCTGGGGTGCGGAGTCT	NM_031678.1	177
CRY1	AAGGGACTCCGGCTGCACGA	CCCCGGATCACAAACAGGCGA	NM_198750.2	203
CRY2	GCCCAGGAGCCACCAAGCAA	GCATGCACACGCAAACGGCA	NM_133405.2	190
CLOCK	ATGTCAGCACAGGCCAGCACA	TGCTCGGCGTCTGGTTTGGA	NM_021856.2	123
BMAL1	ACACTGCACCTCGGGAGCGA	CGCCGAGCTCCAGAGCACAA	AB012600.1	100
REV-ERB*α*	ACAGCTGACACCACCCAGATC	CATGGGCATAGGTGAAGATTTCT	M25804.1	101
Adiponectin	AATCCTGCCCAGTCATGAAG	CATCTCCTGGGTCACCCTTA	NM_144744.3	215
AdipoR1	CGACAGGCCTAAGTGTCCAT	CTTACCCTTCTCCTCCAGCA	NM_207587.1	224
AdipoR2	TGGGAAGTTTTGTTCCTTGG	GCAAGGTAGGGATGATTCCA	NM_001037979.1	201
GAPDH	GTGCCAGCCTCGTCTCATAG	CGTTGATGGCAACAATGTCCA	NM_017008.4	122

**Table 2:** Sperm parameters following exposure to prolonged light or prolonged darkness.

	Control	Light	Dark	ANOVA *F* value (d*f* = 2, 21)
Sperm cell concentration (ml × 125 × 10^4^)	95.625 ± 1.419^a^	141.375 ± 1.619^b^	103.75 ± 1.866^ac^	13.899, *P* < 0.001
Sperm motility (%)	86.428 ± 0.923^a^	93.571 ± 0.250^b^	89.285 ± 0.563^a^	3.421, *P* < 0.05
Sperm abnormalities (%)	20.285 ± 0.966^a^	16.571 ± 0.283^b^	13.857 ± 0.805^c^	10.22, *P* < 0.001

Mean values with different superscript letters are significantly different (*P* < 0.05); d*f*, degree of freedom.

## Data Availability

The data used to support the findings of this study are available from the corresponding author upon request.
